# Autonomous optimization of non-aqueous Li-ion battery electrolytes via robotic experimentation and machine learning coupling

**DOI:** 10.1038/s41467-022-32938-1

**Published:** 2022-09-27

**Authors:** Adarsh Dave, Jared Mitchell, Sven Burke, Hongyi Lin, Jay Whitacre, Venkatasubramanian Viswanathan

**Affiliations:** 1 Department of Mechanical Engineering, Carnegie Mellon University, Pittsburgh, PA 15213 USA; 2 Wilton E. Scott Institute for Energy Innovation, Carnegie Mellon University, Pittsburgh, PA 15213 USA; 3 Department of Materials Science and Engineering, Carnegie Mellon University, Pittsburgh, PA 15213 USA

**Keywords:** Batteries, Mechanical engineering, Software, Electrical and electronic engineering, Materials for energy and catalysis

## Abstract

Developing high-energy and efficient battery technologies is a crucial aspect of advancing the electrification of transportation and aviation. However, battery innovations can take years to deliver. In the case of non-aqueous battery electrolyte solutions, the many design variables in selecting multiple solvents, salts and their relative ratios make electrolyte optimization time-consuming and laborious. To overcome these issues, we propose in this work an experimental design that couples robotics (a custom-built automated experiment named "Clio”) to machine-learning (a Bayesian optimization-based experiment planner named "Dragonfly”). An autonomous optimization of the electrolyte conductivity over a single-salt and ternary solvent design space identifies six fast-charging non-aqueous electrolyte solutions in two work-days and forty-two experiments. This result represents a six-fold time acceleration compared to a random search performed by the same automated experiment. To validate the practical use of these electrolytes, we tested them in a 220 mAh graphite∣∣LiNi_0.5_Mn_0.3_Co_0.2_O_2_ pouch cell configuration. All the pouch cells containing the robot-developed electrolytes demonstrate improved fast-charging capability against a baseline experiment that uses a non-aqueous electrolyte solution selected a priori from the design space.

## Abstract (web summary)

Human-operated optimization of non-aqueous Li-ion battery liquid electrolytes is a time-consuming process. Here, the authors propose an automated workflow that couples robotic experiments with machine learning to optimize liquid electrolyte formulations without human intervention.

## Introduction

High-performance batteries are crucial to the electrification of transportation and aviation^1,2^. However, new battery designs can require extensive manual testing for material optimization, which can take years. Designing a material is fundamentally a complex function that takes material formulation as input and outputs performance. Efficient optimization of such a black-box function via machine learning has been successfully demonstrated in many engineering domains, including catalytic materials^3,4^, photovoltaics^5,6^, solid-state materials^7,8^, and battery-charging protocols^9^. There is a great deal of recent research on coupling automated experiments to these machine-learning methods^10–14^. The hope is that “closed-loop” approaches (i.e., the automated execution of experiments coupled directly to an experiment planner, working in tandem to achieve a goal without human operator influence) display the following traits when compared to the standard design of materials via human-operated experimentation: (1) closed-loop experiments are able to discover optimal material designs within a given design space; (2) closed-loop experiments discover optima faster and with fewer experiments; (3) closed-loop experiments offer a principled basis for design-of-experiments (DOE), balancing exploiting design regions likely to have optimal performance with exploring regions of unknown performance. These traits have been demonstrated in related fields^3,6–8,10,11,15^, but have not yet been demonstrated in battery material design outside of aqueous electrolytes^16^.

Out of the materials present in a battery, liquid electrolytes are a particular challenge to optimize. There are many choices for solvent^17–19^ or salt^20,21^, each potentially yielding vastly different performance; optimized electrolyte solutions often contain more than three or four components. Both species choice and relative proportions of species matter, creating a high-dimensional design space spanning both efficient and inadequate battery performance. Battery electrolytes can be optimized for different applications. The electrolyte design often must fulfill multiple, competing objectives within each application^22^, so optimal designs for rate-capability can differ from optimal designs for cycle-life. Relevant to this work, fast-charging battery electrolytes must be able to transport lithium ions to and into the negative electrode active material at high current rates (5–10 mA/cm^2^), which is strongly associated with bulk transport properties (ionic conductivity, viscosity, diffusivity, cation transference number) and electrode interfacial kinetics (charge transfer impedance, desolvation dynamics)^23,24^.

In this work, we develop a robotic platform named “Clio”, capable of closed-loop optimization of nonaqueous Li-ion electrolyte solutions. Clio enables high-throughput experiments characterizing transport properties over a range of solvents and salts. When connected to an experiment planner, Clio can efficiently and autonomously explore and optimize an objective over a given design space. We consider optimization for fast-charging, focusing initially on single objective optimization of the bulk ionic conductivity as an objective for improving battery rate-capability performance. While this aspect is a preliminary objective function, the workflow introduced in this paper can also enable effective multi-objective optimization^25^ of electrolytes in future studies. Clio autonomously optimized conductivity over solvent mass fraction and salt molality in a design space featuring: ethylene carbonate (EC), ethyl-methyl carbonate (EMC), and dimethyl carbonate (DMC) as a ternary solvent combination; and lithium hexafluorophosphate (LiPF_6_) as a single-salt system. Optimal electrolytes are passed through a sequence of fast-charging electrochemical tests conducted in graphite∣∣LiNi_0.5_Mn_0.3_Co_0.2_O_2_ pouch cells. These results are reported against a baseline electrolyte selected a priori from the design space.

## Results and discussion

**Fig. 1:**
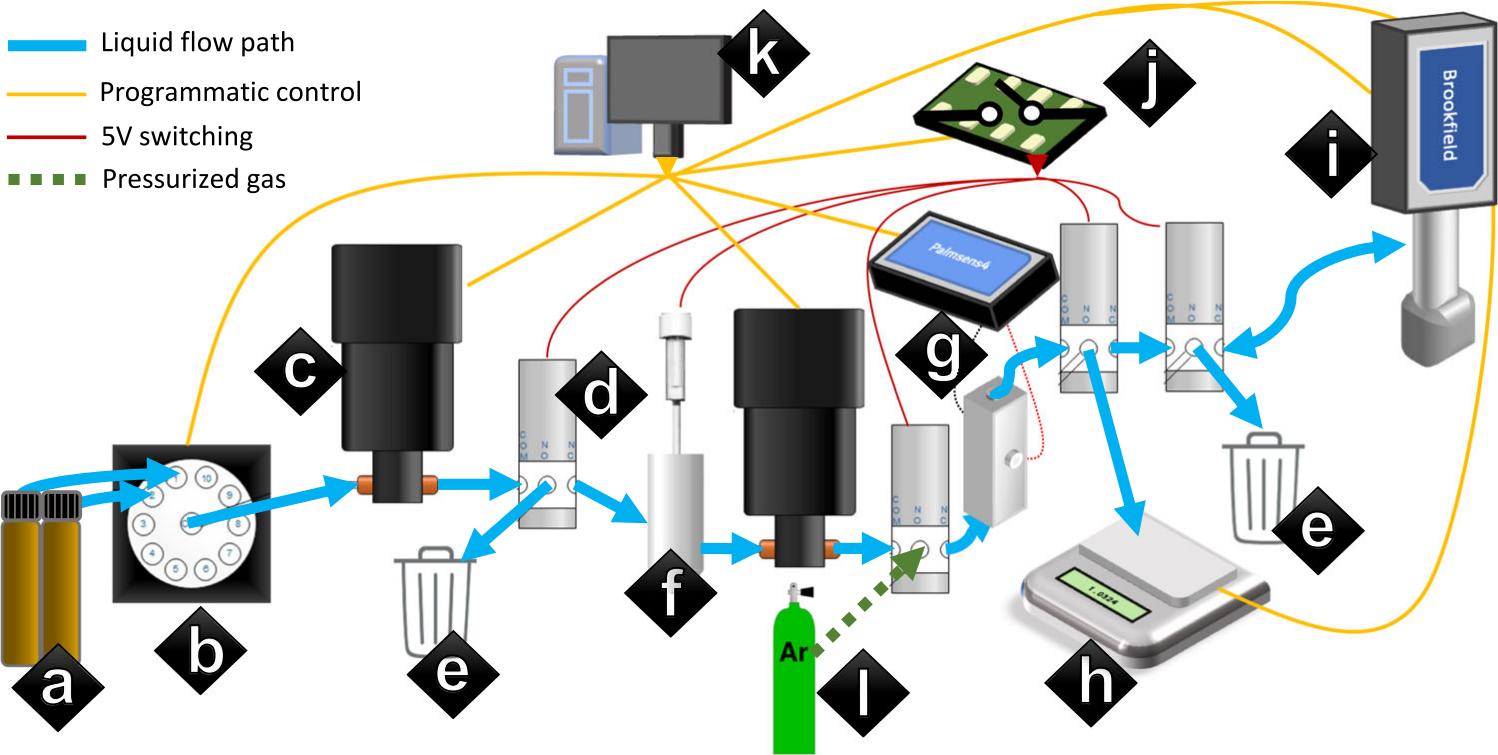
Schematic diagram of automated electrolyte experiment—“Clio”. It uses a series of two programmatic pumps to dose and transfer a liquid sample. Dosing occurs from feeder solutions (**a**) through a 24-port valve (**b**) mediated by pumps (**c**) and a three-way valve (**d**) into either a waste vessel (**e**) or a common vessel with a sonicator for mixing (**f**). Transfer takes the liquid sample through a dual Pt-wire conductivity chamber connected to a Palmsens4 potentiostat (**g**), a three-way valve leading to a mass balance (**h**), and a Brookfield viscometer (**i**). All 5 V switching is handled by a Devantech relay (**j**). Custom Labview software (**k**) orchestrates all instruments. Argon from the glovebox is piped in at high pressure (**l**) to assist in clearing out the closed volume.

Clio is illustrated schematically in Fig. 1, and pictured in Supplementary Fig. 1. The instrument was custom-built for autonomous operations by the authors of the present article and is essentially an automated liquid handler at the front end (inspired by previous lab automation^26^), and a flow-through liquid electrolyte characterization tool at the back end. Given a specified electrolyte, Clio will create it by mixing together feeder solutions and measure the ionic conductivity, viscosity, and density of the liquid sample. The instrument is a closed volume with all wetted surfaces comprised of polytetrafluoroethylene (PTFE), stainless steel, or platinum (Pt). This geometry requires a solvent rinse cycle between each composition tested. Acetonitrile was used as a rinse solvent due to its provision of high vapor pressure, low viscosity, and high LiPF_6_ solubility^27–29^. The orchestration for the rinse cycle has been open-sourced (see “Code availability”). All experiments are run in triplicate as developed during a rinse solvent contamination study (Supplementary Fig. 2). Ionic conductivity is measured via electrochemical impedance spectroscopy (EIS) measurements using a Palmsens4 (compact potentio/galvanostat and frequency analyzer made by Palmsens) connected to symmetric Pt wires. The temperature of the sample under test was taken during each ionic conductivity evaluation via thermocouple, and remained between 26 and 28 °C for all measurements reported. The mean absolute difference between repeated measurements of the ionic conductivity of the same electrolyte is found to be ± 1.3% across a range of electrolytes (Supplementary Figs. 3 and 4). Samples can be retained in 2-mL vials for use in characterization outside of Clio; this feature was used to test electrolytes measured to have high ionic conductivity in pouch cells outside of Clio.

The liquid sample was dosed and transferred by two positive-displacement pumps made by Fluid Metering Inc (FMI) calibrated to 5 µl per stroke. Compared to other pump types and test geometries, these valveless, reversible pumps enable multiplexing of many feeder solutions with fewer valves, complexity, and capital expenditure. However, single tracking through a pump leads to challenges in priming the pump and handling viscous solutions, solutions to which are discussed in “Methods”.

LabView was used to orchestrate all devices-specific interfaces are detailed in Supplementary Table 1, and the code is available to the public, segregated into high-level orchestration VIs for runs and rinses and low-level control VIs as specified in the README. The autonomous experiment literature has already explored C++/ROS and Python options^30,31^ for experiment management. LabView was selected for ease of development, management of multiple VISA connections via NI-MAX, and reliably low latency (single milliseconds) for triggering calls to these multiple VISA connections—this last point is crucial for Clio as high-pressure argon contacts many valves, pumps, and instruments in the rinse process. However, LabView is naturally less modular, shareable, and maintainable than the other languages, and lacks version control entirely; thus, it may be worthwhile to further assess LabView against the other frameworks already present. Clio is controlled over HTTP via a LabView REST API, where a client requests experiments from Clio in JSON and Clio returns experimental results. This enables facile integration with any experiment planner. A management layer handling inventory volumes, chemical conversions, grid creation, and HTTP session management was designed in Python—this code is made publicly available. Further details on Clio’s hardware, software, and operation are contained in “Methods”.

A system of ternary solvents featuring EC, EMC, and DMC with LiPF_6_ as salt was chosen for study. This is a well-known space for electrolyte design and an appealing domain to demonstrate a novel experimental method. EC-EMC 30–70% mass fraction with 1.1 mol LiPF_6_ per kilogram solvent was chosen a priori as a baseline, as EMC is commonly chosen as a co-solvent in relevant literature to benchmark electrolyte transport properties^32–34^.

**Fig. 2:**
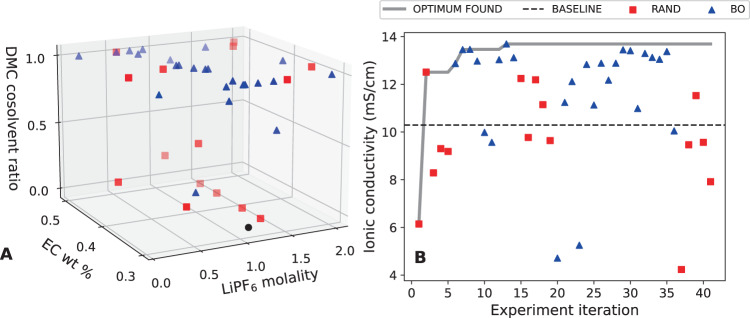
Sampling in an electrolyte design space during optimization. **A** The specific points (electrolytes) sampled by Clio during autonomous optimization of ionic conductivity in an EC-EMC-DMC, LiPF_6_ design space. The black circle indicates baseline electrolyte selected a priori (EC/EMC 30%/70% by mass, 1.1 m LiPF_6_). **B** Learning rate of Clio during this optimization. Random sampling (RAND) is interspersed with Bayesian optimization (BO), biasing the optimization toward exploring the space. Dashed black line indicates the conductivity of the baseline electrolyte as measured by Clio.

**Fig. 3:**
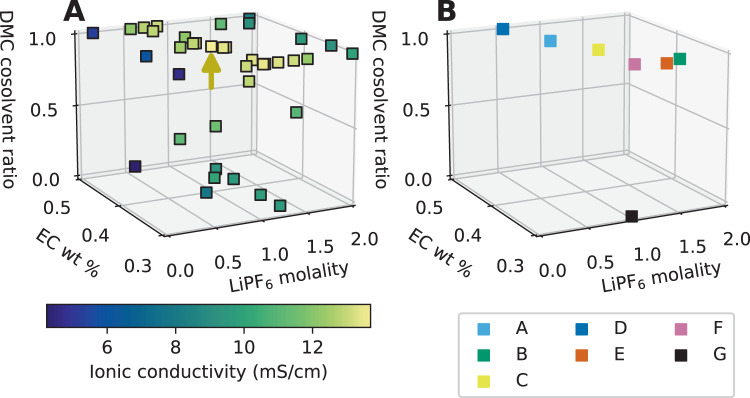
Evaluations of the electrolyte design space, and electrolytes chosen for cell testing. **A** Measured ionic conductivities (in color) of each electrolyte sampled by Clio during autonomous optimization of ionic conductivity in an EC-EMC-DMC, LiPF_6_ design space. The gold arrow indicates the highest ionic conductivity electrolyte found (EC/DMC 40%/60% by mass, 0.9 m LiPF_6_). **B** Electrolyte candidates chosen for testing in pouch cells. Blends A–F are discovered by Clio. Blend G is the baseline electrolyte.

Dragonfly—an open-source Bayesian optimization package designed for black-box optimization^25,35^—is used as the experiment planner in this work. Dragonfly’s adaptive sampling strategy and broad support for discretized and constrained domains were of interest to the authors of the present article. The experiment domain was represented to Dragonfly by three axes: (1) EC mass fraction, (2) DMC co-solvent ratio, calculated as (mass fraction of DMC)/(1 − mass fraction of EC), and (3) LiPF_6_ molality. Axis 1 was limited to EC mass fractions of 30% to 50%, and axis 2 was limited between 0 and 2 mols LiPF_6_ per kilogram solvent, keeping with conventional choices for non-aqueous liquid electrolyte design. Each axis was split into 10–12 equivalently spaced levels, creating more than 1000 points within the domain to search. Dragonfly optimized for ionic conductivity over 42 experiments. This optimization campaign was initialized with five samples drawn randomly from a space-constrained to the top and bottom faces of the design space (i.e., EC-EMC and EC-DMC electrolytes only)—subsequent random sampling was not subject to this constraint. The electrolytes investigated are illustrated as points in the three-dimensional design space in Figs. 2 and 3.

An adaptive DOE is constructed with three different acquisition functions evaluated over a Gaussian process regression-based surrogate model—expected improvement, top-two expected improvement, and upper-confidence bound. These sampling algorithms roughly balance exploration and exploitation. We interspersed the optimization run with periodic random sampling to further favor exploration. Figure 2 illustrates a convergence on an ionic conductivity optimum within 15 experiments. The optimizer evaluates mainly in the high DMC region compared to the high EMC region, generally in the middle band of salt concentration between 0.7 to 1.3 mols LiPF_6_ per kilogram solvent (known to be near the concentration of peak conductivity in standard non-aqueous Li-ion electrolyte solutions). Samples from the high and low salt, mixed DMC-EMC regions could also be desired.

Figure 3 shows measured ionic conductivities in the three-dimensional design space. Linear carbonates like DMC are mixed with cyclic carbonates like EC to lower viscosity and increase ionic conductivity^22^, but more recent studies reveal that ionic conductivity can also increase with higher dielectric constant as it improves ion dissociation (e.g., EC over DMC, DMC over EMC)^36^. Our study indicates that the ionic conductivity optimum of the EC:DMC:EMC LiPF_6_ system at 26–28 °C is found at EC:DMC 40:60 by mass, 0.9 m LiPF_6_ with 13.7 mS cm^−1^ (gold arrow in Fig. 3). Further experiments were conducted on the EC:DMC face of the design space along the 30%, 40%, and 50% by EC mass contours, confirming this optimum in this design space (Supplementary Figs. 5 and 6). Theoretical calculations conducted with the Advanced Electrolyte Model (AEM; a highly accurate model for nonaqueous electrolyte transport properties^37,38^) also show a higher ionic conductivity in the 40% EC blend compared to 30% or 50%—this could be due to the improved ion dissociation in the 40% compared to the 30%, and lowered viscosity in the 40% relative to the 50% (Supplementary Fig. 7).

Out of 42 evaluations, seven candidate electrolytes were picked for follow-up Li-ion cell assembly and testing. Three of the highest ionic conductivity electrolytes were chosen (blends A, C, and F in Fig. 3), as were two higher-salt concentration electrolytes with >10 mS/cm (blends B and E) and one lower-salt concentration electrolyte with >10 mS/cm (blend D). As mentioned above, EC-EMC 30–70% by weight, LiPF_6_ 1 m was a baseline electrolyte denoted blend G. The cell-testing candidates are given with their color codes in Table 1. A suite of tests on small pouch cells was devised to test electrolyte candidate for fast-charging capability. Dry cells of 220 mAh capacity were received from the manufacturer with electrode materials, current collectors, and tabs pre-made. Detailed cell specifications, materials, and procedures are given in Methods. To guard against cell-to-cell discrepancies, all cells were run in at least pairs. Candidate electrolytes were mixed by Clio and routed into the retain rack (blue tray, bottom left of Supplementary Fig. 1). A human operator emptied Clio’s retain rack and manually injected each electrolyte into these dry cells.

**Table 1:**
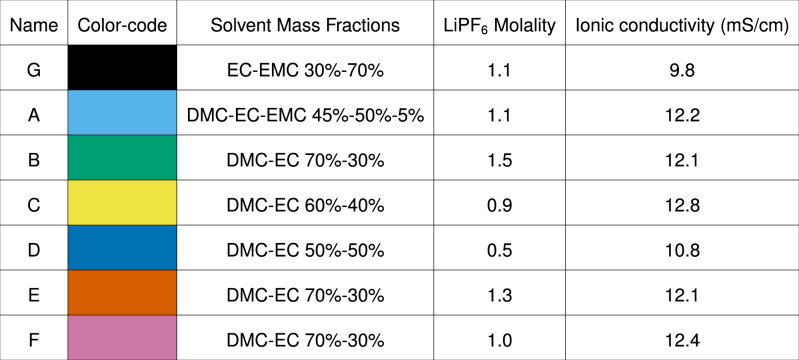
Table of electrolyte compositions involved in autonomous optimization

Blend C and F had the highest measured ionic conductivities in the space; blend B and E had high salt concentrations and >10 mS/cm ionic conductivity; blend D had a low salt concentration and >10 mS/cm. Blend A had the highest ionic conductivity with EMC present. Blend G was chosen as a baseline electrolyte a priori.

Cells were assembled and preconditioned, then subject to a five-step rate test of increasing constant-current charges up to 10.4 mA/cm^2^ (4C), with 1.3 mA/cm^2^ (0.5 C) constant-current discharges between each step, each followed by constant-voltage (CV) hold at 4.3 V until a termination current of 0.7 mA/cm^2^ (C/20). Cells were then repeatedly cycled at the highest charge rate until failure due either to overvoltage (>4.5 V) or capacity fade. Further details on this cell testing—including current conversions and cell assembly, preconditioning, and materials—are given in “Methods” and Supplementary Table 2.

**Fig. 4:**
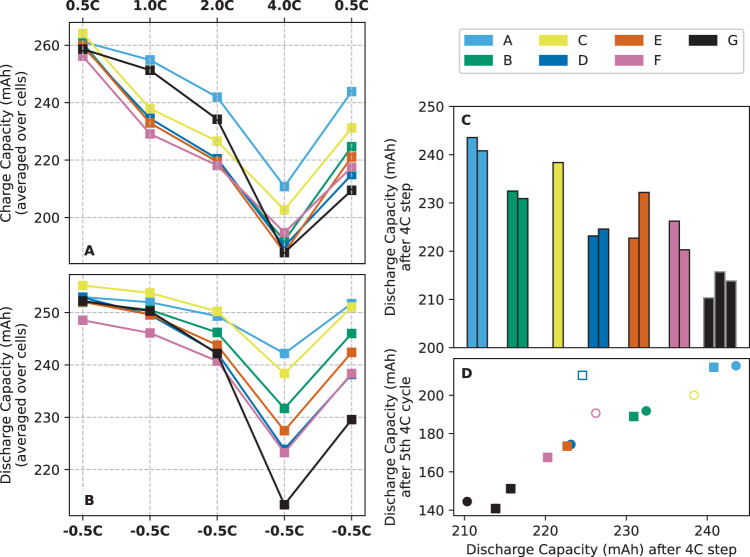
Li-ion cell performance with various electrolyte solutions. **A** Constant-current charge capacity of each electrolyte, averaged from two cells. **B** Constant-current discharge capacity of each electrolyte, averaged from two cells. **C** Bar chart of each cell's discharge capacity after 4 C charge—cycle 7 on panels **A** and **B**. **D** Relationship between performance at 4 C rate-test step and capacity of cell after five back-to-back 4 C charge cycles; unfilled markers are cells that did not make it five cycles—the last cycle capacity is reported here.

Figure 4A–C gives the results from the rate-test for each candidate electrolyte. Electrolyte blends optimized by Clio show greater or equal discharge capacity at the 4C step compared to baseline, showing greater usable capacity put into the cells at this high charging rate (Fig. 4B). The worst cell containing a Clio electrolyte showed a 5% improvement on discharge capacity after 4C charging compared to the worst baseline cell, and the best cell with a Clio electrolyte showed a 13% improvement on this metric compared to the best baseline cell (Fig. 4C). Capacity fade during these further 4C charge cycles, along with coulombic efficiency and related metrics, are shown in Supplementary Fig. 8. Discharge capacity after the 4C step of the rate-test shows correlation with discharge capacity after five cycles of 4C charging (Fig. 4D).

These results indicate that our workflow is able to optimize material designs within a given design space, and deliver an optimum from a common design space. Better fast-charging performance over baseline in a pouch cell was realized after only 42 experimental evaluations optimizing for bulk ionic conductivity. Similar closed-loop workflows have been demonstrated in the literature to discover optimal materials faster or with fewer experiments compared to random or exhaustive searches^8,11,39,40^. These are frequently assessed with two figures-of-merit with respect to random sampling—an enhancement factor (the relative improvement in discovered optima over samples taken) and acceleration factor (the relative improvement in samples required to achieve a given value of the objective)^12,41^. We calculate these quantities with simulated optimizations over the design space multiple times, using a machine-learning model as the “black-box function” to be optimized. This model was fit to data calculated by AEM, and shows great fidelity when predicting on points sampled by Clio (Supplementary Fig. 9).

**Fig. 5:**
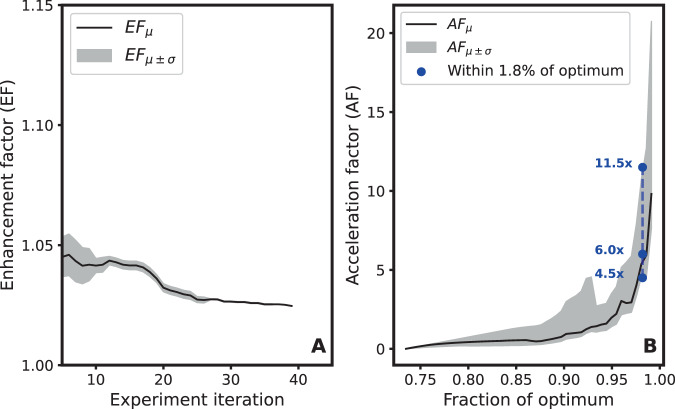
Calculation of enhancement factor (EF) and acceleration factor (AF). These are calculated based on the simulated optimizations shown in Supplementary Fig. 10. Standard deviations are calculated from the spread of outcomes of the random sampling baseline. **A** EF remains upper-bounded by 5% over the optimization. **B** AF achieves a value of 10 on average for finding the true optimum, and a value of 6 at 1.8% of optimum (this is near Clio's experimental precision).

Campaigns comparing various forms of Bayesian optimization to random sampling are shown in Supplementary Fig. 10, out of which we calculate enhancement and acceleration factors (EF, AF, respectively). For the form of optimization used in the fast-charging study, EF (Fig. 5A) is upper-bounded by 5%, trending to 2.5% over 40 samples. This may be due to design space, as shown in Supplementary Figure 11: the objective has a single optimum and varies quite smoothly with wide bands of high conductivity throughout the space. The AF (Fig. 5B) reaches 10× on average for at the optimum objective—again, due to the smooth, widely dispersed objective in this design space, AF is negligible until 95% of the maximum objective. Relevant to Clio and its precision in measuring conductivity, the AF for achieving a 98.5% of the maximum objective is on average 6x and ranges from 4.5×–11.5× depending on individual runs.

An automated workflow also adds time and material efficiency compared to human testing, detailed in Supplementary Table 3 and Supplementary Fig. 12. For repeated conductivity measurements on two electrolyte samples, Clio likely performs more measurements per day than an average human operator and uses about 30% of the volume compared to literature equivalents. Still, this conclusion suggests that future work aim to improve the acceleration factor of autonomous experiments as physical automation tends to yield fixed efficiency improvements that scale, at best, linearly with sample size. Research efforts on applying improved machine-learning methodologies to plan experiments in complex design spaces may unlock greater overall efficiencies than focusing on laboratory automation alone. Already, literature has demonstrated AFs above 20× to 1000×^11,12,41^, implying the potential for higher AFs when Clio searches complex design spaces.

In summary, we have demonstrated a “closed-loop” optimization of a non-aqueous battery electrolyte for ionic conductivity and close the device gap to show performance improvements when tested in pouch-cell configuration. Our workflow using a Bayesian experiment planner produces a highly efficient DOE, finds a yet-unreported conductivity optimum in a well-studied design space, and reveals candidates with better fast-charging performance in a Li-ion cell than an intuitively chosen baseline. This demonstrates the potential of closed-loop experiments to discover optimal material designs within well-explored and unexplored design spaces. By comparing this workflow in simulation to a randomly sampled optimization, we estimate a six-fold overall acceleration using our scheme. We believe this work will be useful beyond the battery community; our custom-designed robotic platform, experiment planning, and integration with device testing will be valuable in optimizing other autonomous discovery platforms for energy applications and material science in general.

## Methods

### Clio: hardware and metrology

Hardware utilized in Clio’s design is seen in Fig. 1 and Supplementary Table 1. The flow path of the liquid sample is determined two pumps (Fluid Metering Inc. ICST-02) and three three-way valves (Cole–Parmer) that are switched, along with a Qsonica Q55 sonicator (55 watt, 20 kHz) in the mixing vessel, by a Devantech relay. Crucial to this work, ionic conductivity is measured using electrochemical impedance spectrocopy (EIS) in a custom chamber, a PTFE fixture in which the liquid sample fills between symmetric platinum electrodes. Complex impedance is measured at five frequencies between 14 and 800 kHz—the real part of the impedance at the frequency with the smallest measured phase difference is taken as the resistance of the sample. A cell constant is derived from single point calibration to an acetonitrile solution of known ionic conductivity (see Supplementary Fig. 2)—inverse resistance divided by cell constant is reported as the specific ionic conductivity of the sample.

A rinse cycle is required between samples, for which we chose acetonitrile. The contamination study in Supplementary Figure 2 shows that, after a rinse, two experiments will converge on the known conductivity of a standard. All experimental evaluations are run in triplicate for this reason, with the final two runs in each triplet averaged and reported as the measurement. The repeatability of the conductivity measurement is assessed in Supplementary Figs. 3 and  4—120 samples were taken in triplicate across a range of carbonate solvents and LiPF_6_ concentrations. These were assessed for the variance between run 2 and run 3 in each set, finding a mean averaged percent error of 1.3% and a 95% confidence interval of ± 3.8%.

The temperature of the sample under test was taken during each conductivity evaluation via a thermocouple connected to the Palmsens4 (18-bit resolution combined potentiostat, galvanostat, and frequency response analyzer), and remained between 26 and 28 °C for all measurements reported. Clio is kept in a glovebox maintaining a dry argon atmosphere, with moisture levels measured every 5 min to be below 10 parts per million H_2_O and oxygen levels remaining below 100 parts per million.

### Clio: viscous solution handling, priming

Two challenges of using a single valveless pumps to manage dosing and transfer include accurately dosing solutions of wide viscosity ranges and priming the pump. As viscosity increases, the pump must slow down its strokes per minute (RPM) to maintain its calibrated microliter-per-stroke value. This is illustrated in Supplementary Fig. 13—solutions of known densities and viscosities are pumped through the dosing pump at a variety of RPMs. The viscosimeter used was a Brookfield DV-II Pro (cone and spindle viscosimeter, ± 1% full-range accuracy model RVDV-II+ with RV2 spindle). By massing the output, we derive a microliter-per-stroke value and compare that to the calibrated setting. The pumps have a stated precision of 0.5% around the calibration mark; cutoffs for each viscosity level were set conservatively based on this data. The resulting linear curve is shown in Supplementary Fig. 14. This curve is highly dependent on tubing diameter and system pressure—the priming tube had an internal diameter of 1.016 mm, and the feeder vials feature a small hole (~1.5 mm diameter) in the lids to minimize pressure differential within the vials. These choices enabled a top-end dosing viscosity of 70 centipoise for Clio as set up in this study, and further optimizations for higher viscosity design spaces can be discussed in future work. For a study focusing on ionic conductivity, transfer pump precision is less important. We simply used a 50 stroke offset from the dosing pump derate curve to account for the higher system pressure in the back half of Clio (marker g–j in Fig. 1). More precise derating of the transfer pump will feature in future work on viscosity, density, and surface tension measurements.

The pumps used in this study only operate at high accuracy when they are filled with fluid—a state of being primed. Thus, in the dosing process, the pump must change from primed (dosing solution 1) to unprimed to primed again (dosing solution 2). This requires an open-air valve maintained in the VICI valve (marker b in Fig. 1), and a three-way valve switching between waste and the mixing vessel (markers d, e, and f, respectively, in Fig. 1). Furthermore, the authors of the present article developed a priming method that alternates between feeder solution and open-air valve to de-contaminate shared lines (present in dose_FMI.vi) in a volume-efficient manner. When pulling the feeder solution through the unprimed pump, the RPM was slowed down by a factor of 3, which was visually verified to prime the pump at the viscosity range present in the derate curve.

### Clio: software

Clio is orchestrated with a suite of Labview tools developed in-house, focusing on low-level device communication and timings. Labview manages a webhook for supplying Clio a specific DOE; electrolyte compositions are passed to the webhook via HTTP, and Clio’s measurements are passed back in the response. Experiment and inventory management is handled through a Python API that interfaces with this webhook; any generic machine-learning recommender can interface with this given appropriate configurations. Two Python classes are central to Clio operation. "Experiment” objects manage Clio sessions, inventory, and input/output with the Labview control suite. Because Clio mixes by volume, "ElectrolyteComposition” objects handle electrolyte representation and conversions needed to transform electrolyte-composition axes (molality and mass fractions in this work) to volumes of feeder solutions. An additional script is provided that creates grids of volumes across *n* feeder solutions and converts the grid to composition axes in a fast, vectorized manner.

Electrolyte conversion also requires a priori estimates of feeder solution density; these values were estimated via the Advanced Electrolyte Model^37,42^, a high-fidelity electrolyte calculation software, and confirmed by Clio’s density measurement. All code used is made available, see “Code availability”. The client managing Clio experiments sits on an AWS EC2 instance managed by Toyota Research Institute.

### Clio: advanced electrolyte model

As mentioned, the Advanced Electrolyte Model^37,42^ (AEM) was used for a variety of uses throughout this study. AEM is a proprietary electrolyte calculation software providing estimations of transport, thermodynamic, and other electrolyte quantities at high fidelity across a large swath of liquid electrolytes relevant to battery operation. In this study, we used densities and viscosities from AEM in dosing and derating liquid samples in Clio. We also used data from AEM in the simulated optimizations further discussed below.

### Clio: machine learning

Dragonfly—the code-base used for Bayesian optimization in this study—is open source and available at github.com/dragonfly/dragonfly.

The “live” optimization are run by wrapping Clio’s *Experiment* object into Dragonfly’s *maximise_function* API. The run was initialized with 5 random samples pulled from top and bottom faces of the design space cube (either DMC or EMC co-solvent, not a mixture)—this traded off random sampling with a space-filling design to seed optimization. Dragonfly’s Bayesian optimization (BO) default settings were used for the live run—a Gaussian process regressor formed by the Matern kernel was used with parameters fit every experiment round with maximum likelihood estimation (MLE). Dragonfly uses an adaptive choice of acquisition function^35^. The agent starts optimization by sampling a uniform distribution containing acquisition functions upper-confidence bound (UCB), expected improvement (EI), and top-two expected improvement (TTEI). If the experiment run with the acquisition function drawn from this sample improves the optimum found, the distribution is incremented in favor of this acquisition function.

We examined the acquisition function dependence of the optimization in simulated runs. These simulated runs were obtained by drawing “experiment” data from a Gaussian process regressor trained on data from the Advanced Electrolyte Model (AEM). This regressor was also formed by a Matern kernel and fit by MLE, with the simulated objective shown in Supplementary Fig. 11. Results for these simulated optimizations are seen in Supplementary Fig. 10, averaged over 10 runs (except for random sampling which is run 120 times). We see that all BO-inclusive campaigns perform much better than random sampling in discovering the optimum, and do not show strong dependence on initialization as each campaign ends up discovering the design space optimum. Acquisition function dependence is also weak, as performance differences are negligible between adaptive, UCB, and TTEI-led campaigns. Often, acquisition function choice is crucial to good performance^35^, but this does not appear to be the case in this simple design space. Enhancement factor (EF) and acceleration factor (AF) were calculated in an identical manner to recent literature^11,12,41^. Standard deviations are calculated from spread of outcomes of the random sampling baseline, as this variance is much larger than variance in the Bayesian optimization strategies.

### Materials availability

The solvents and solutes used in this investigation, except for the baseline electrolyte and acetonitrile, were obtained as anhydrous (<20ppm moisture), battery-grade (99.9% pure) materials from GELON LIB Group. Materials used for pouch-cell testing were also obtained from the GELON LIB Group. Baseline electrolyte of 1.1 m LiPF_6_ in EC:EMC 30:70 and acetonitrile was obtained from Sigma-Aldrich, anhydrous (<10 ppm) and battery-grade (99.9% pure).

Stock feeder solutions were made for this investigation through first making solute-free mixtures of solvents, then gradually adding the appropriate mass of solute. All solutions were mixed for a minimum of 30 min past the dissolution of the last visible solute. All measurements of solvent and solute were done by mass using a Denver Instrument PI-214.1 analytical balance (210 g capacity, 0.1-mg readability/repeatability). All solutions were mixed with a VWR brand magnetic stir bar and magnetic stir plate. All glassware and stir bars were washed thoroughly with acetonitrile between solutions and were allowed to dry completely before any more solutions were made. Miscibility and co-solubility screening tests were conducted on these feeder solutions before solutions were used in the test stand. Solutions were routinely checked for the stability of dissolved species and were routinely inverted to prevent any stratification of the solutions. Feeder solutions were stored in 60-ml amber glass vials with Sure/Seal septa lids. All materials were stored and handled in a dry argon atmosphere with <5 ppm moisture. All experimental procedures, except cell testing, were also conducted in a dry argon atmosphere.

### Cell testing

Dry pouch cells with a nominal capacity of 220 milliampere-hours (mAh) were sourced from Linyi Gelon LIB Co. Ltd. Cells contained LiNi_0.5_Mn_0.3_Co_0.2_O_2_ cathode and graphite anode. Cells were run in duplicate or triplicate—blend C was only run once due to a cell presenting with incorrect capacity. Full information on cell materials, dimensions, compositions, and specifications is given in Supplementary Table 2.

All tests were completed on Neware BTS-CT-4008-5V12A battery cyclers. Testing was carried out in an uncontrolled temperature environment (i.e., no environmental/climactic control). The average lab temperature was 25 °C ( ± 2 °C). Cells were cut open and dried in a vacuum oven overnight and then transferred into a glovebox, with moisture levels below 10 parts per million H_2_O and oxygen levels remaining below 100 parts per million. Cells were then filled with 0.7 mL of electrolytes by an Eppendorf pipette and sealed with a heat sealer. Finally, cells were transferred out for the following four steps done in sequence: (1) cell construction followed by a 36-h rest held at 1.5 V for electrolyte infiltration, (2) cell formation at C/20 (1C = 2.6 mA/cm^2^) symmetric for three cycles, (3) rate-test with charge rates of C/2, 1C, 2C, 4C, then C/2 again, discharging each time at C/2. Each constant-current charge was completed with a constant-voltage hold at 4.3 V until C/20. Each C/2 discharge was completed with a constant-voltage hold at 2.5 V until C/20. Then, each cell was subjected to a cycling test until failure—either overvoltage of 4.5 V during the charge or low capacity. Each cycle had a charge rate of 4C, discharge rate of 0.5 C, completing each cycle again with a CV hold at 4.3 V. Each C/2 discharge was completed with a constant-voltage hold at 2.5 V. Cells were rested for 10 min between each step of the test. An image of an example dry pouch cell with a length scale, identical to the cells used in this study, is provided in Supplementary Fig. 15. Translations of C-rates into currents, specific currents, and current densities are given in Supplementary Table 4.

## Supplementary information

Supplementary Information

## Data Availability

All data generated in this study have been deposited in Github for public accession at github.com/BattModels/Clio-NatCommData.

## Code availability

All code generated in this study have been deposited in Github for public accession at github.com/BattModels/Clio-LabViewControl (for LabView orchestration code) and github.com/BattModels/Clio-PythonAPI (for Python management API).
