# Supplementary material for: Autonomous optimization of non-aqueous Li-ion battery electrolytes via robotic experimentation and machine learning coupling
Source: Nat Commun. 2022 Sep 27;13:5454. doi: 10.1038/s41467-022-32938-1 (PMC9515088; doi:10.1038/s41467-022-32938-1)
Supplement: Supplementary file 1 — Supplementary Information [file 41467_2022_32938_MOESM1_ESM.pdf]

Supplementary Information for Article, "Autonomous optimization of non-aqueous Li-ion battery electrolytes via robotic experimentation and machine learning coupling"

Adarsh Dave[a,b], Jared M. Mitchell[b,c], Sven Burke[b,c], Hongyi Lin[a,b], Jay Whitacre[b,c], and Venkatasubramanian Viswanathan[a,b,*]

[a] *Department of Mechanical Engineering, Carnegie Mellon University, Pittsburgh, Pennsylvania, 15213, USA.*

[b] *Wilton E. Scott Institute for Energy Innovation, Carnegie Mellon University, Pittsburgh, Pennsylvania, 15213, USA.*

[c] *Department of Materials Science and Engineering, Carnegie Mellon University, Pittsburgh, Pennsylvania, 15213, USA.*

[*] *Email: venkvis@cmu.edu*

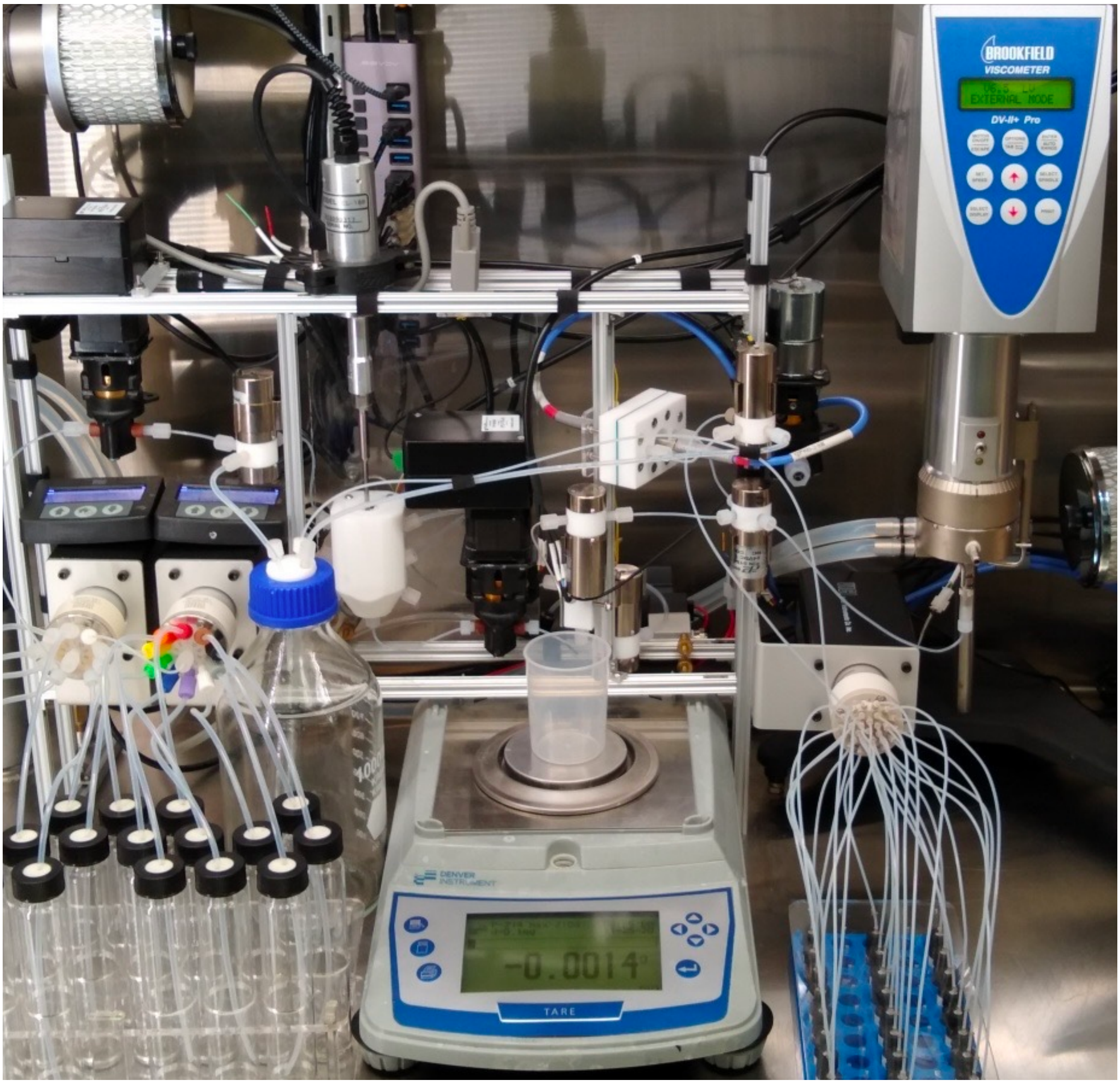

Supplementary Figure 1: Image of "Clio" as utilized in this study.

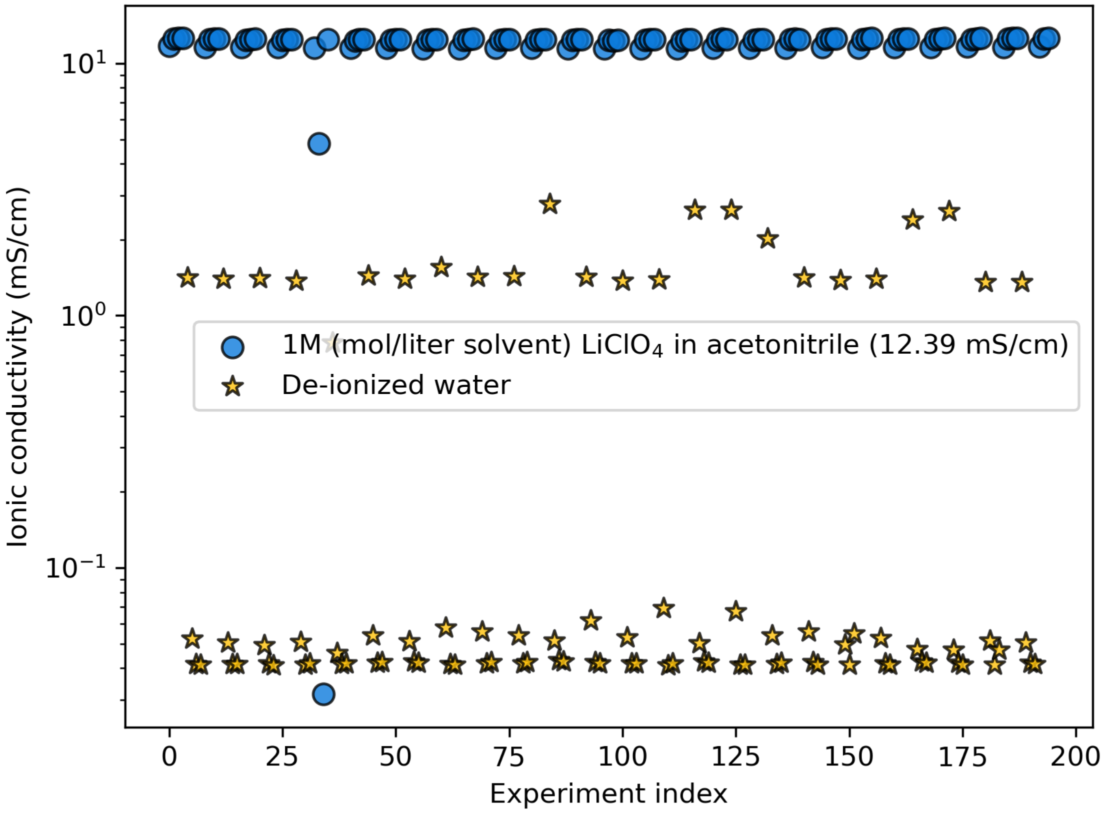

Supplementary Figure 2: Contamination study of extended operation of Clio. Clio alternates between conductivity standard (here, acetonitrile and $LiClO_4$ with conductivity of 12.39 mS/cm) and de-ionized water, before the glovebox was put under a controlled environment. Running experiments in triplicate ensures two uncontaminated evaluations per electrolyte. One experiment of $LiClO_4$ in acetonitrile failed due to running out of feeder solution (near index 30)

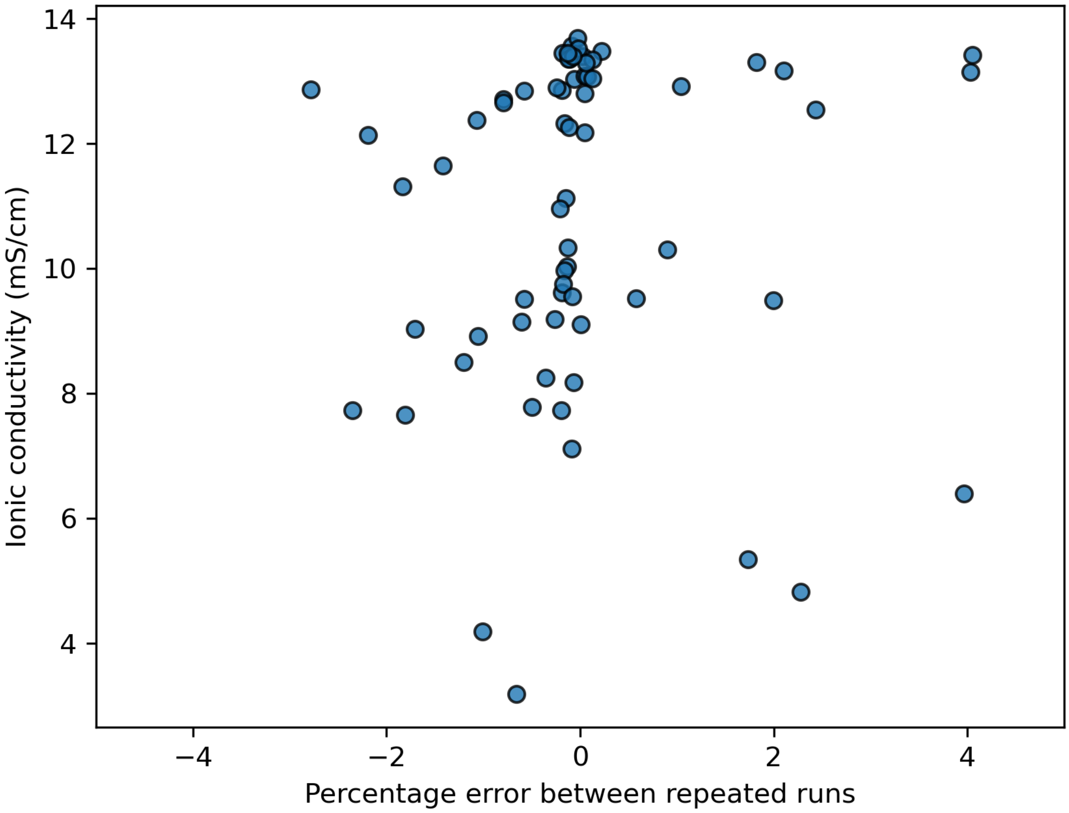

Supplementary Figure 3: Percentage error between repeated evaluations of the same electrolyte, for a range of 67 electrolytes within the EC-DMC-EMC-DEC + $LiPF_6$ design space. Error appears uncorrelated with conductivity level. Temperature varied between 26-28 °C throughout runs, and only on average 0.5% within repeats.

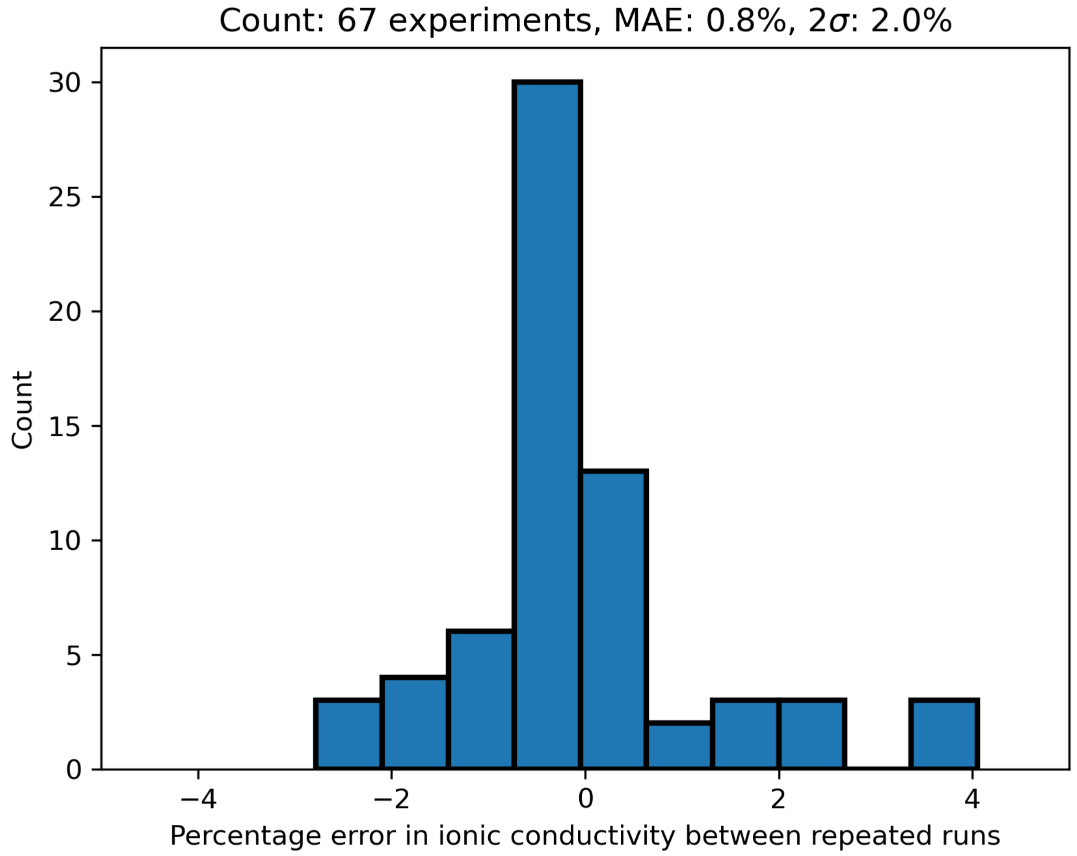

Supplementary Figure 4: Percentage error between repeated evaluations of the ionic conductivity of the same electrolyte, for a range of 67 electrolytes within the EC-DMC-EMC-DEC + $LiPF_6$ design space. Mean absolute percentage error is 0.8%, with a 95% confidence interval of 2.0% error. Temperature varied between 26-28 °C throughout runs, and only on average 0.5% within repeats.

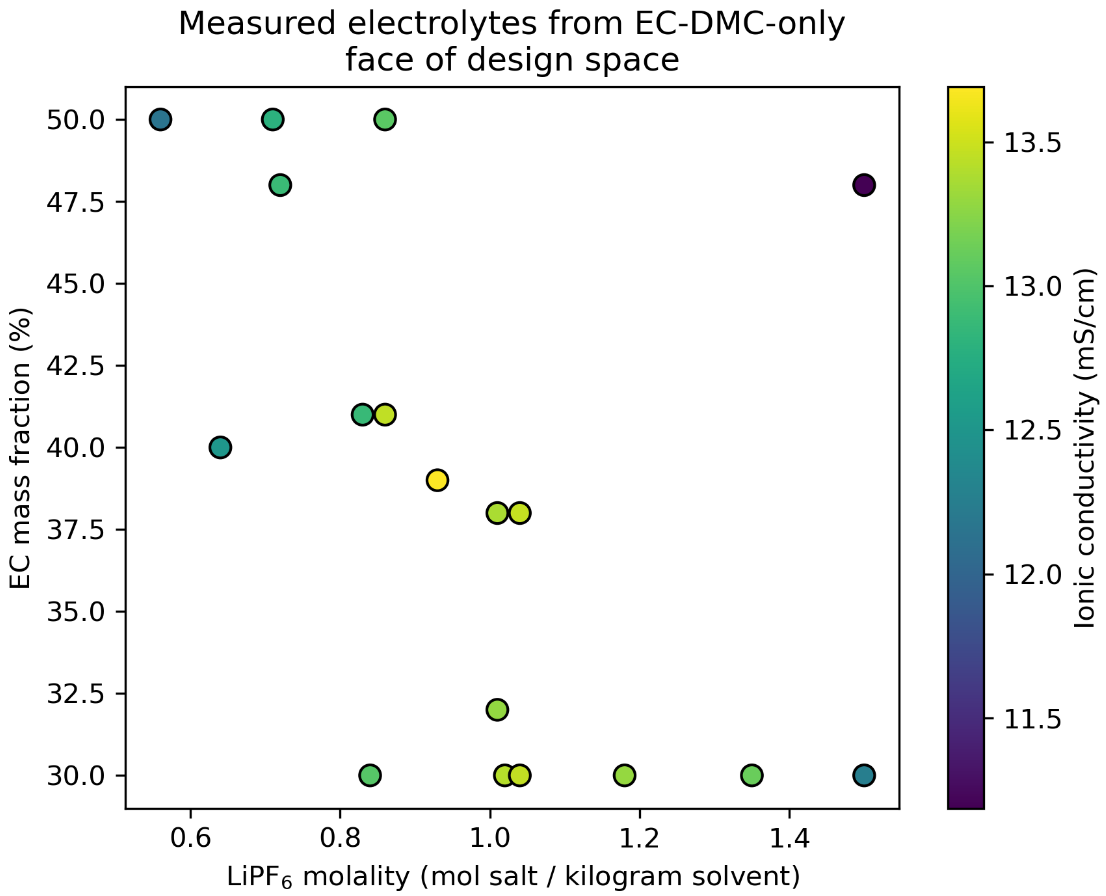

Supplementary Figure 5: Electrolytes sampled during optimization from the top-face of the design space - electrolytes only containing EC and DMC (no EMC). This region showed the greatest ionic conductivity in the design space. The experiments indicate a maximum of conductivity at 26-28°C near 40% EC mass fraction.

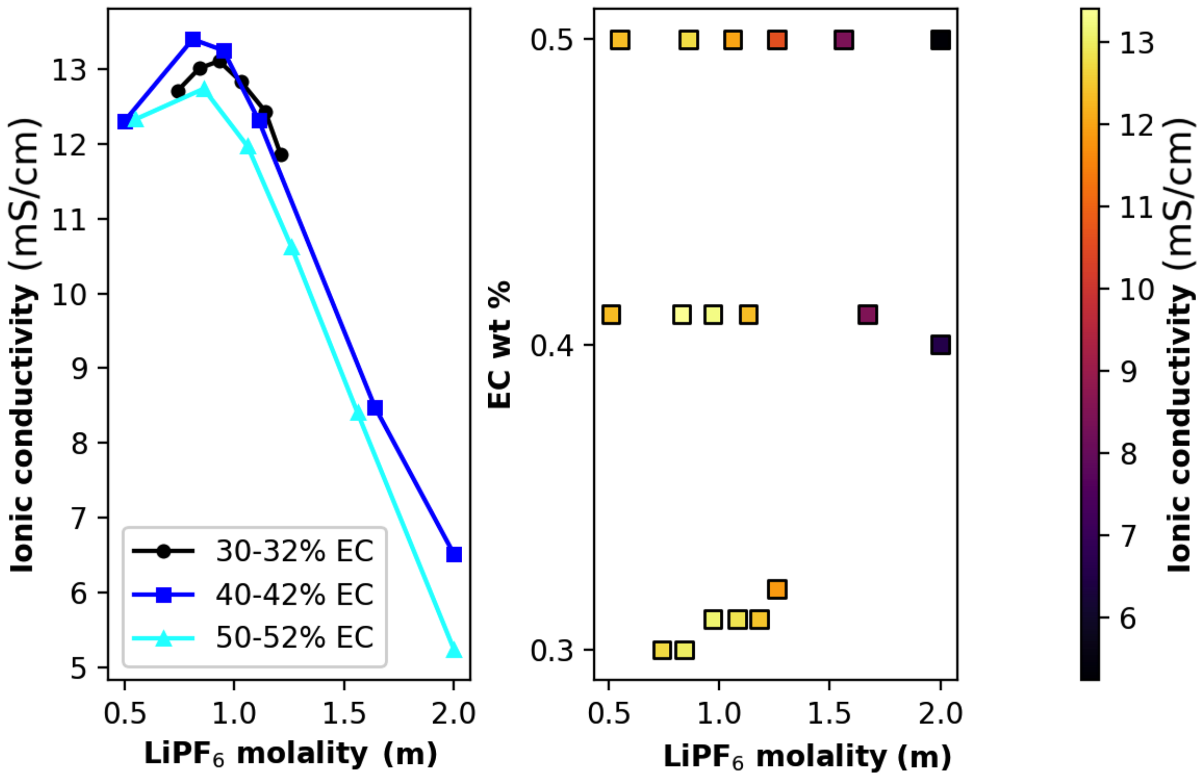

Supplementary Figure 6: Re-surveyed contours on the EC and DMC only face of Figure 2a - due to a small error in density calculation, contours run were within 2% of each EC mass fraction level. These data show that the maximum of conductivity at 26-28°C again appears to be near 40% EC mass fraction rather than 30%.

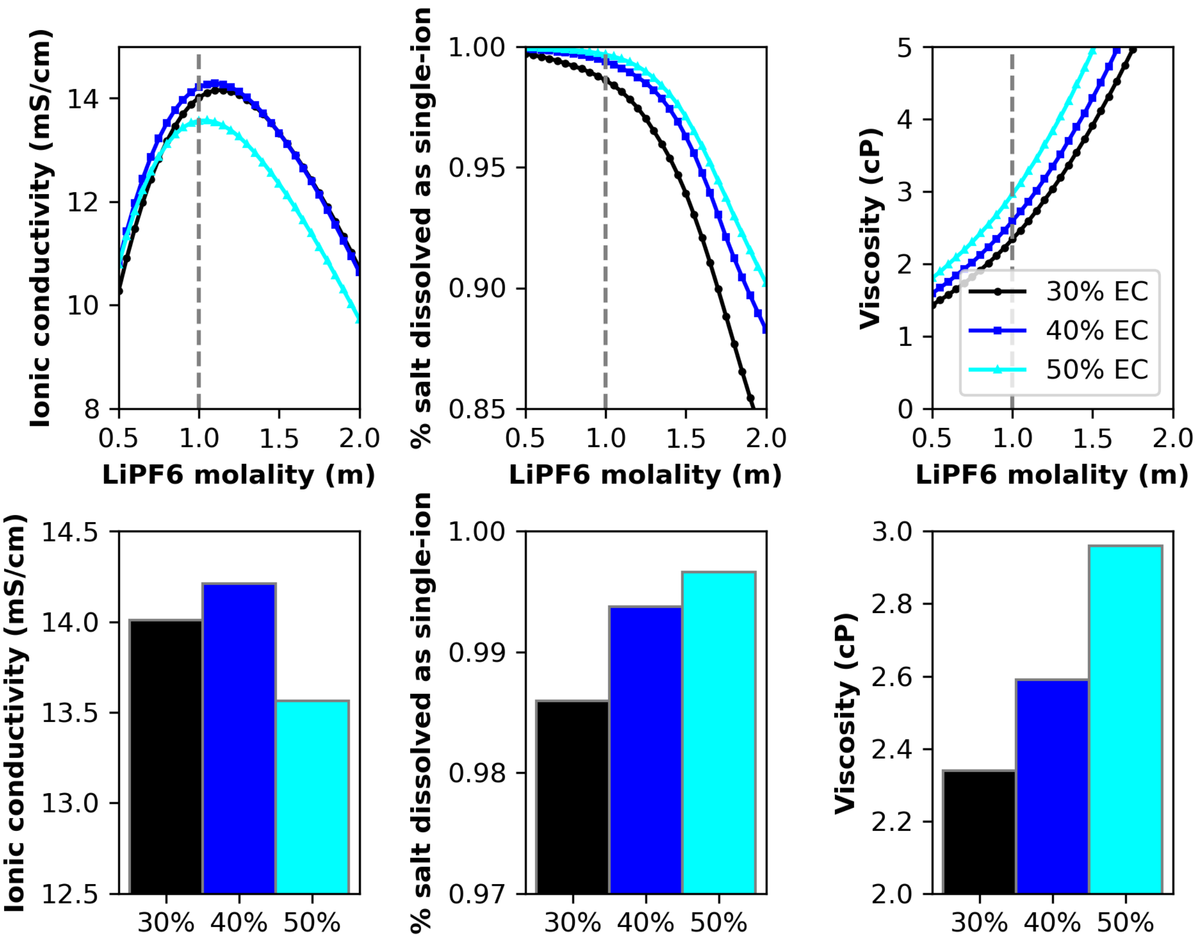

Supplementary Figure 7: Conductivity and two covariates obtained from Advanced Electrolyte Model for EC:DMC $LiPF_6$ at 30°C. The model corroborates findings that the maximum ionic conductivity in the system at this temperature is near 40% EC, though experiments find a higher difference in peak conductivity at a lower molality. Two explanatory covariates - percentage of salt dissolved as single-ions and viscosity - are posited. At 1m $LiPF_6$, 40% EC mass fraction shows single-ion population similar to 50% EC, but a viscosity more similar to 30% EC, potentially leading to the increased ionic conductivity.

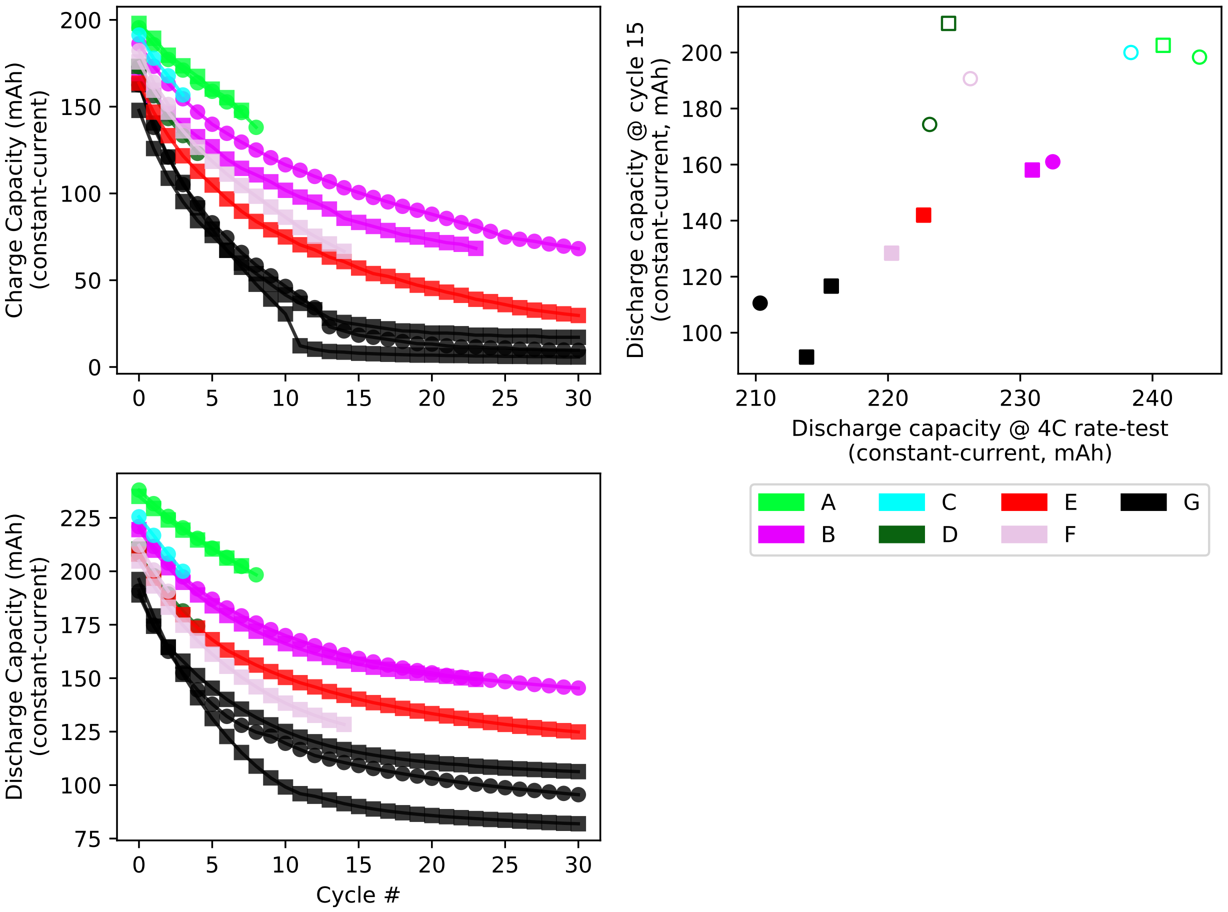

Supplementary Figure 8: Cycling data for each Li-ion pouch cell during repeated 4C charges, 0.5C discharges over 30 cycles.

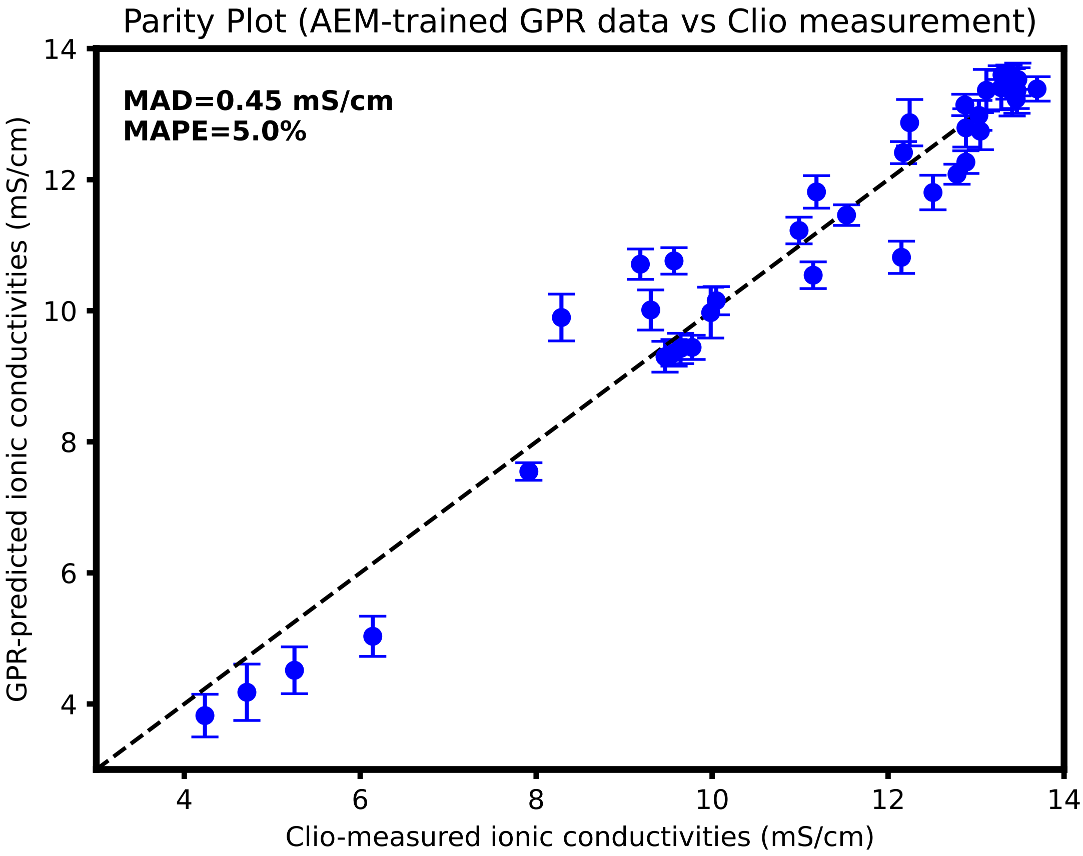

Supplementary Figure 9: A Gaussian Process regression model was trained on Advanced Electrolyte Data in the same space as the "live" optimization. This model can be used to predict the same points Clio sampled, shown in this parity plot comparing model-predicted points with Clio measurements; given the moderate mean absolute deviation (MAD) and mean average percent error (MAPE), this model describes the design space well and is further used to evaluate acceleration/enhancement of our sampling algorithm against random.

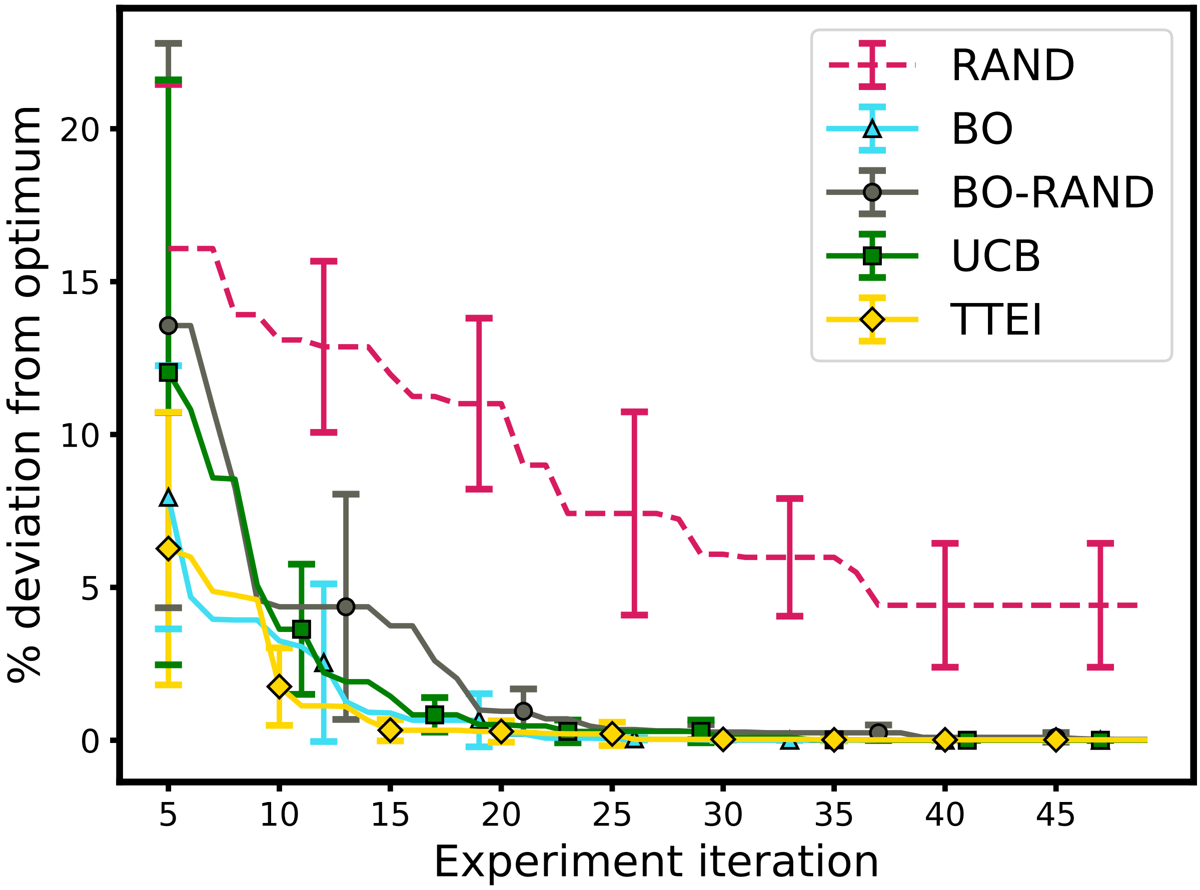

Supplementary Figure 10: Using the machine-learning model described in Supplementary Figure 9, simulated optimizations were conducted with various Bayesian optimization strategies (BO, BO-RAND, UCB, TTEI), and compared with random sampling (RAND). Each point is the average of 10 independent runs (except RAND, which was run 120 times), with error bars showing the standard deviation across these runs. All campaigns were initialized with five random samples. BO represents Dragonfly's default, three acquisition function portfolio approach; BO-RAND additionally intersperses 5 random samples every 15 BO samples. UCB restricts this to solely upper-confidence bound sampling, while TTEI does this for top-two expected improvement only.

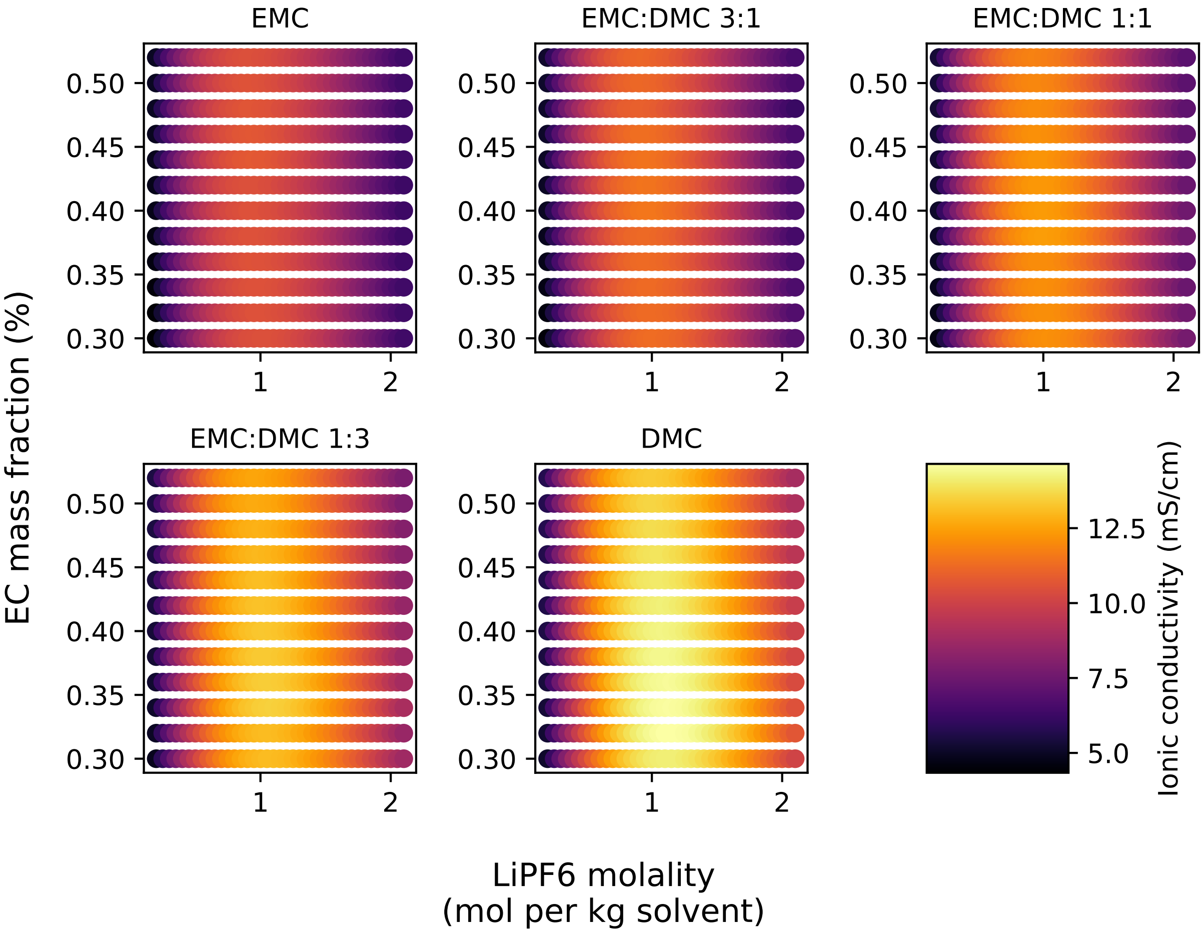

Supplementary Figure 11: Conductivity from the design space in the simulated optimization is provided by the machine learning model discussed in Supplementary Figure 9 - this figure shows these conductivities in the colorspace from the full design space. Each subplot shows a particular value for the DMC co-solvent ratio, with x-axis as $LiPF_6$ molality and y-axis as EC mass fraction.

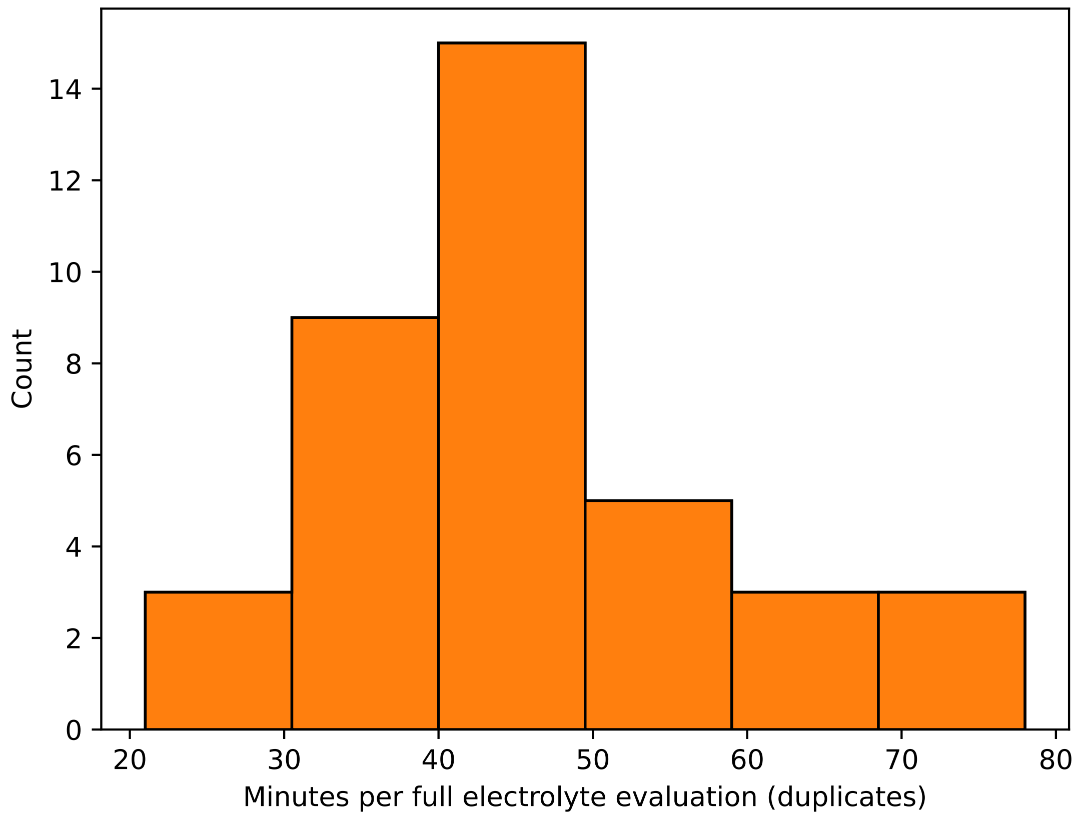

Supplementary Figure 12: This histogram shows the per-electrolyte evaluation time in Clio for this study. This includes two evaluations per electrolyte and cleaning time. The distribution mainly represents variance in the components of each electrolyte - as more feeder solutions are put into a sample, time to dose (thus time to evaluate) will increase.

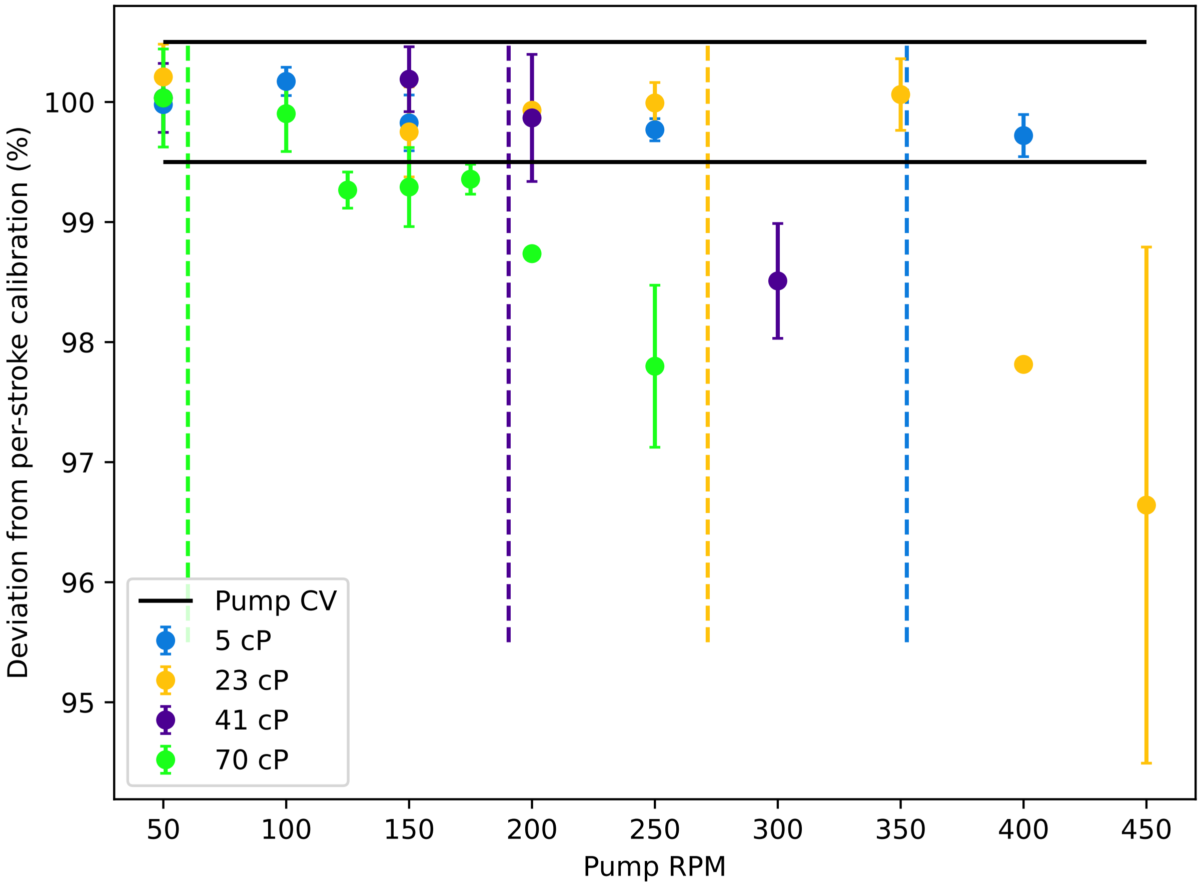

Supplementary Figure 13: The pumps used in this work must run at slower revolutions-per-minute (RPM) when pumping higher viscosity fluids, to maintain their microliter-per-revolution (or stroke) calibration. This figure shows data that were used to calibrate a four-point linear derate used to slow pumps down for a fluid of given viscosity. Given a known pump precision of 0.05% deviation ("Pump CV"), four fluids of various viscosities were tested to see the cutoff RPM that assures pumps remain within their stated precision. This deviation from per-stroke calibration is estimated by allowing the pump to dose fluids of known density onto a mass balance for a given amount of revolutions. The cutoffs are shown in dotted lines of the same color as their corresponding fluid viscosity - these cutoffs are used in the linear derate curve shown in Supplementary Figure 14.

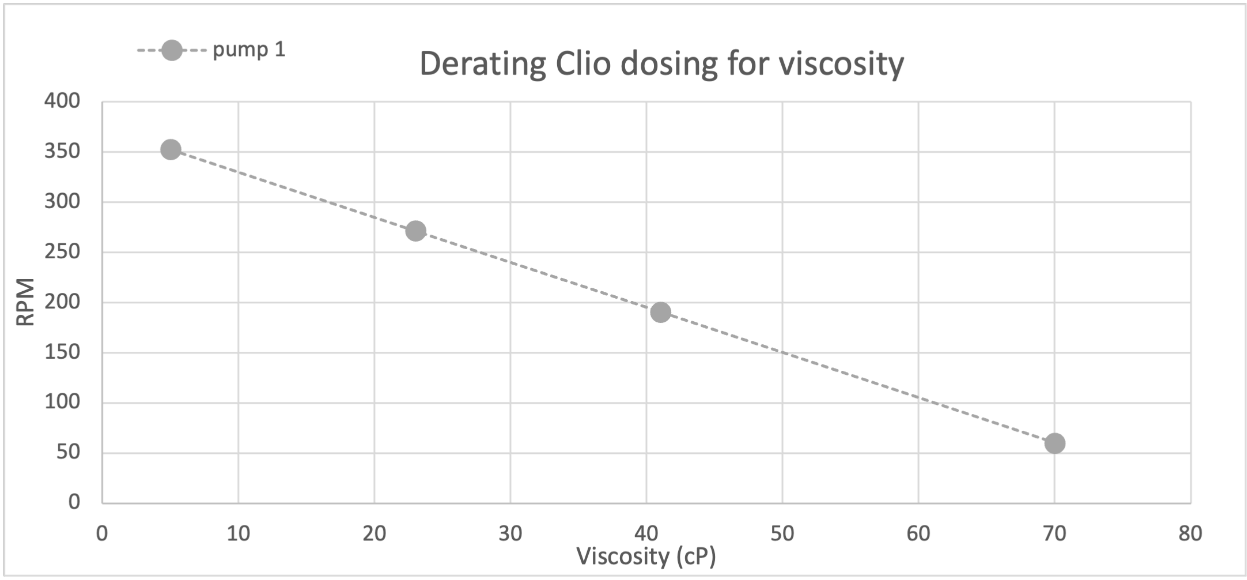

Supplementary Figure 14: The cutoff values for each viscosity level in Supplementary Figure 13 are plotted against their viscosity. This line is used to derate the dosing RPM for a given fluid. This requires a measurement of viscosity of the feeder solution - either the Advanced Electrolyte Model or Clio can provide this data.

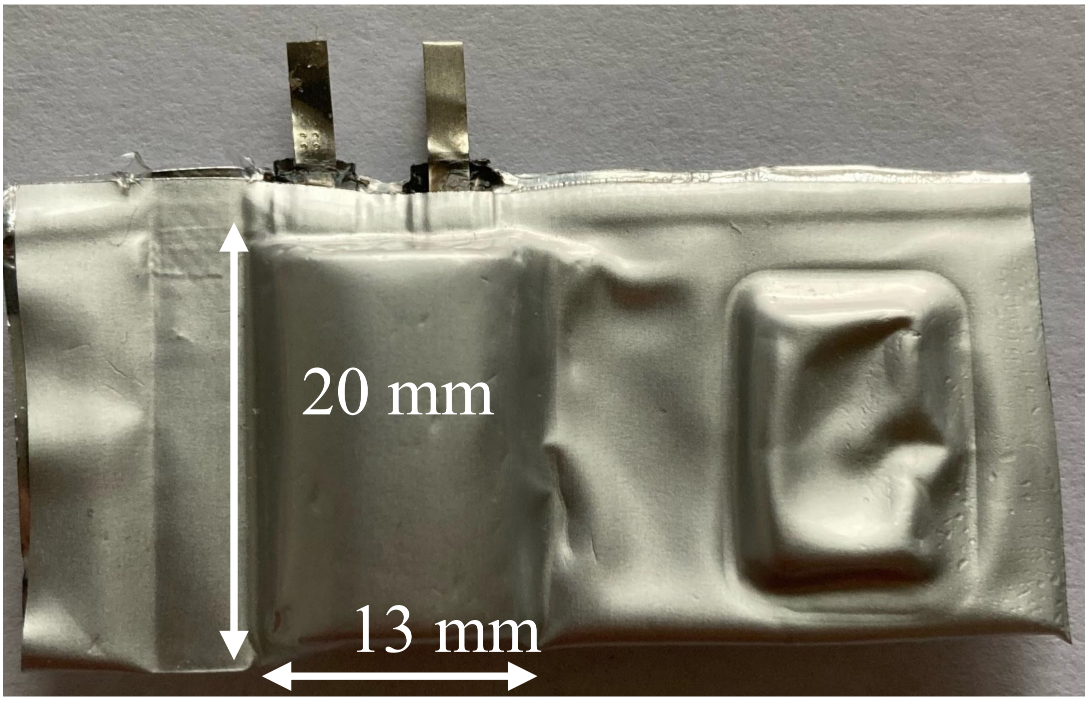

Supplementary Figure 15: Image and scale for dry pouch cells used in the study.

Component	Use	Communication	.vi name
VICI Valco	Valve of feeder solutions	VISA/Serial	*VICI Valve_1*.vi
FMI Pumps	Dosing, transfer of solution	C++ .dll	*FMI_CH1*.vi
Palmsens4	EIS, potentiostat	.bat file	*PS_CMD*.vi
Balance	Mass of known volume	VISA/Serial	*Read_Balance*.vi
Brookfield DVII	Viscometer	VISA/Serial	*VISCOSITY*.vi
DVT Relay	Switching	VISA/Serial	*DVT_RelayBoard*.vi

Supplementary Table 1: Components and integration information for Clio. "DVT" stands for Devantech. "FMI" stands for Fluid Metering Inc. "VICI" refers to Valco Instruments Co. "DVII" refers tothe DV-II+ model viscometer from Brookfield.

**Cathode**	*double-coated, rolled electrode*	
**composition:** NMC532(98% wt), carbon black (1%), PVdF (1%)		
	**total weight (g)**	1.628
	**capacity (mAh/g)**	150
	**length (cm)**	23.176
	**width (cm)**	1.832
	**thickness (µm)**	50
	**coating porosity %**	40
	**total area (cm^2)**	84.92
	**areal loading (mAh/cm^2)**	2.59
**Anode**	*double-coated, rolled electrode*	
**composition:** Graphite(95% wt), CMC (2.5%), SBR (2.5%)		
	**total weight (g)**	0.93
	**capacity (mAh/g)**	335
	**length (cm)**	24.752
	**width (cm)**	1.878
	**thickness (µm)**	60
	**coating porosity %**	30
	**total area (cm^2)**	92.97
**Separator**		
**composition:** polyproplene, single-layer		
	**areal weight (mg/cm^2)**	1.2
	**length (cm)**	59.6
	**width (cm)**	1.942
	**thickness (µm)**	25
	**porosity %**	55
**Cathode CC**	*** current collector**	
	**composition:** aluminum, foil	
	**density (g/cm^3)**	2.7
	**length (cm)**	47.372
	**width (cm)**	1.832
	**thickness (µm)**	15
**Anode CC**	*** current collector**	
	**composition:** copper, foil	
	**density (g/cm^3)**	8.92
	**length (cm)**	52.766
	**width (cm)**	1.886
	**thickness (µm)**	11
**Electrolyte**	*composition varies in study*	
	**volume (mL)**	0.7

Supplementary Table 2: Table giving material parameters for all components of dry pouch cells used in study, sourced from Gelon (see Methods).

	Human	Clio
Minutes per evaluation	18 (12-25)	45 (30-60)
Hours per workday	7-8	24
Evaluations per workday	17-40	24-48
Test-volumes (mL)	20[1]	6

Supplementary Table 3: Comparison for evaluation time, volumes, and throughput for human-operated experiments vs. Clio automation

C-rate	C/20	C/2	1C	2C	4C
mA	11	110	220	440	880
mA/g	33.8	67.6	135.1	270.3	540.5
mA/cm^2	0.65	1.3	2.6	5.2	10.4

Supplementary Table 4: Conversions for all currents mentioned in this study. “mA” denotes milliamperes.

Supplementary References

[1] Ma, X. *et al.* A study of highly conductive ester co-solvents in Li[Ni0.5Mn0.3Co0.2]O2/Graphite pouch cells. *Electrochim. Acta* **270**, 215–223 (2018). URL `https://www.sciencedirect.com/science/article/pii/S0013468618304948`.
